# Understanding Calcium-Mediated Adhesion of Nanomaterials in Reservoir Fluids by Insights from Molecular Dynamics Simulations

**DOI:** 10.1038/s41598-019-46999-8

**Published:** 2019-07-24

**Authors:** Hsieh Chen, Shannon L. Eichmann, Nancy A. Burnham

**Affiliations:** 1 Aramco Services Company: Aramco Research Center-Boston, Cambridge, MA 02139 USA; 2 Aramco Services Company: Aramco Research Center-Houston, Houston, TX 77084 USA; 3 Physics and Biomedical Engineering Departments, Worcester Polytechnic Institute, Worcester, MA 01609 USA

**Keywords:** Atomistic models, Nanoscale materials

## Abstract

Interest in nanomaterials for subsurface applications has grown markedly due to their successful application in a variety of disciplines, such as biotechnology and medicine. Nevertheless, nanotechnology application in the petroleum industry presents greater challenges to implementation because of the harsh conditions (i.e. high temperature, high pressure, and high salinity) that exist in the subsurface that far exceed those present in biological applications. The most common subsurface nanomaterial failures include colloidal instability (aggregation) and sticking to mineral surfaces (irreversible retention). We previously reported an atomic force microscopy (AFM) study on the calcium-mediated adhesion of nanomaterials in reservoir fluids (S. L. Eichmann and N. A. Burnham, *Sci. Rep*. 7, 11613, 2017), where we discovered that the functionalized and bare AFM tips showed mitigated adhesion forces in calcium ion rich fluids. Herein, molecular dynamics reveal the molecular-level details in the AFM experiments. Special attention was given to the carboxylate-functionalized AFM tips because of their prominent ion-specific effects. The simulation results unambiguously demonstrated that in calcium ion rich fluids, the strong carboxylate-calcium ion complexes prevented direct carboxylate-calcite interactions, thus lowering the AFM adhesion forces. We performed the force measurement simulations on five representative calcite crystallographic surfaces and observed that the adhesion forces were about two to three fold higher in the calcium ion deficient fluids compared to the calcium ion rich fluids for all calcite surfaces. Moreover, in calcium ion deficient fluids, the adhesion forces were significantly stronger on the calcite surfaces with higher calcium ion exposures. This indicated that the interactions between the functionalized AFM tips and the calcite surfaces were mainly through carboxylate interactions with the calcium ions on calcite surfaces. Finally, when analyzing the order parameters of the tethered functional groups, we observed significantly different behavior of the alkanethiols depending on the absence or presence of calcium ions. These observations agreed well with AFM experiments and provided new insights for the competing carboxylate/calcite/calcium ion interactions.

## Introduction

Using nanomaterials for subsurface applications, such as oil and gas recovery, reservoir imaging, flow diagnostics, CO_2_ sequestration, and environmental remediation, has attracted great attention in recent years^1–11^. The unique properties of nanomaterials have the potential to achieve much higher performance in the above mentioned applications. For example, the ultra-sensitive optical properties^12,13^ (e.g. persistent luminescence and surface-enhanced Raman scattering) may be used in reservoir interwell tracers for improved waterflood recovery optimization^14–16^. In addition, the distinctive interfacial properties^10^ (e.g. wedge-film disjoining and Pickering emulsion formation) may be used to mobilize residual oil given the correct tailoring of the nanomaterial chemistry and concentration. Unfortunately, harsh subsurface environments with high salinity (>120 000 ppm of total dissolved solids) and often elevated temperatures (>100 °C) impose great engineering challenges for the applications for nanomaterials in these conditions^17–26^. Specifically, the colloidal stability of nanomaterials is largely compromised by excessive charge screening and subsequent loss of electrostatic repulsion at the high salinities^3,4,24–26^. In addition, the dehydration and collapse of steric polymer coatings at high temperatures renders most common nanomaterial stabilizers unusable in subsurface conditions^3,4,24–26^. In addition to nanomaterial-nanomaterial aggregation, nanomaterial-mineral surface adsorption leads to irreversible retention and represents another great challenge that must be addressed^3,4,17–23^. Material loss through irreversible retention correlates directly to the costs of applying these materials. Classical approaches and theories (e.g. DLVO theory) have been applied in this area with limitations. Clearly, a new paradigm is needed to solve the above challenges before we can successfully deploy nanomaterials for advanced subsurface applications.

The concept of specific ion effects has been recognized for decades, yet the microscopic details, physical origins, and potential implications have been controversial, leading to significant debate in the scientific community^27–39^. Recent adhesion force measurements using atomic force microscopy (AFM) demonstrated interesting ion-specific behavior of nanomaterials in reservoir fluids^40^. In this study, carboxylate-functionalized AFM tips (and to a lesser extent the oxidized bare silicon tips) showed decreased adhesion to calcite surfaces in calcium (Ca^2+^) ion rich fluids. Moreover, this effect was insensitive to the total ionic strength (salinity) of the fluid, which contradicted classical DLVO theory predictions. Another AFM study similarly demonstrated that the adhesion force increases with increasing hydration radius through specific ion effects when carboxylate groups are present^41^.

**Figure 1:**
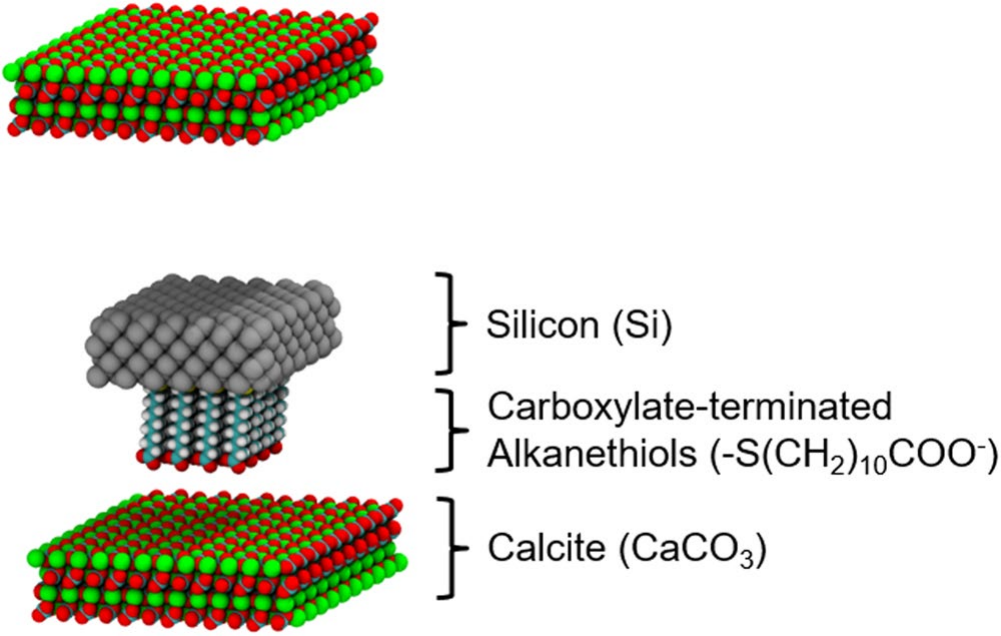
Schematic of simulation setup. The atom colors are: Ca = green; C = cyan; O = red; H = white; S = yellow; Si = gray.

**Figure 2:**
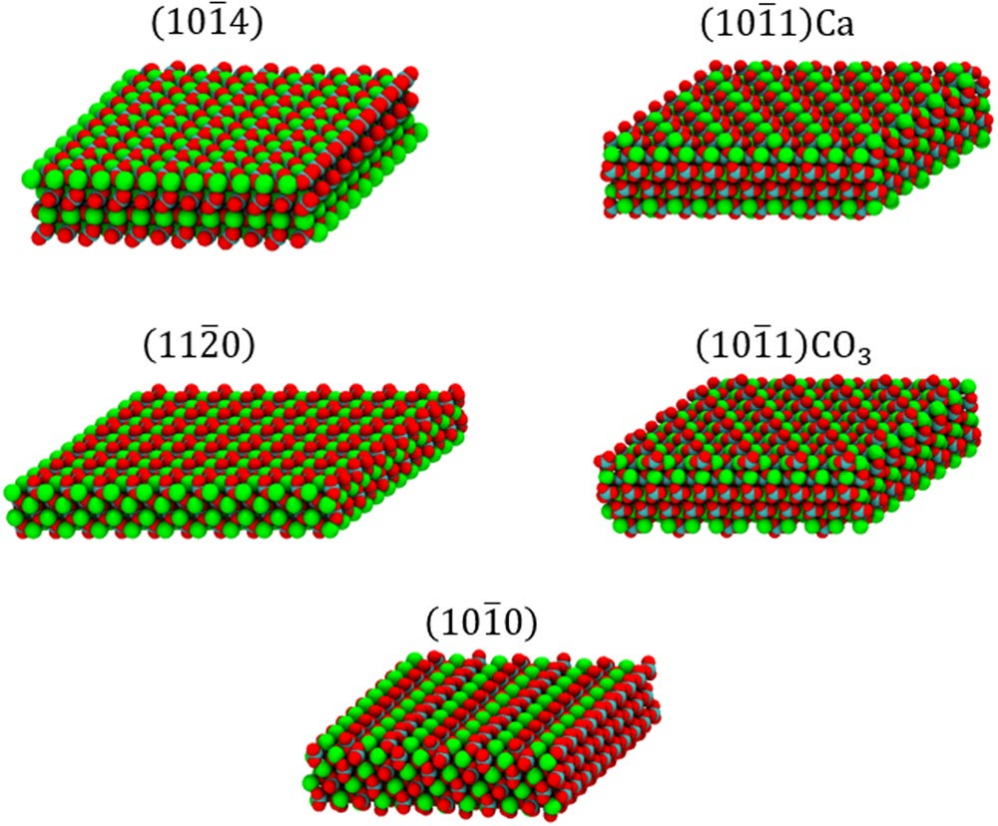
Snapshots of different calcite crystallographic surfaces. The atom colors are the same as in Fig. 1.

In the present work, we aim to provide molecular-level understanding of the AFM experiments by means of atomistic molecular dynamics (MD) simulations. These simulations focus on the carboxylate-functionalized tips (Fig. 1) because of the strong ion-specific effects of these functional groups. As we will show, the MD results precisely captured the experimental trends. In addition, the simulations revealed the fine balance between the carboxylate-ion, carboxylate-calcite, and calcite-ion interactions in different reservoir fluids on diverse calcite crystallographic surfaces (Fig. 2). By analyzing the order parameter of the alkanethiols tethered to the AFM tips, we further discovered that their behavior throughout the adhesion measurement was affected by the presence of specific ions. We believe the molecular insights put forward in this study may have important implications for stabilizing nanomaterials in harsh subsurface oil and gas reservoirs, or other similar conditions found in the environment, biology, and pharmaceuticals^42,43^.

## Results and Discussion

**Figure 3:**
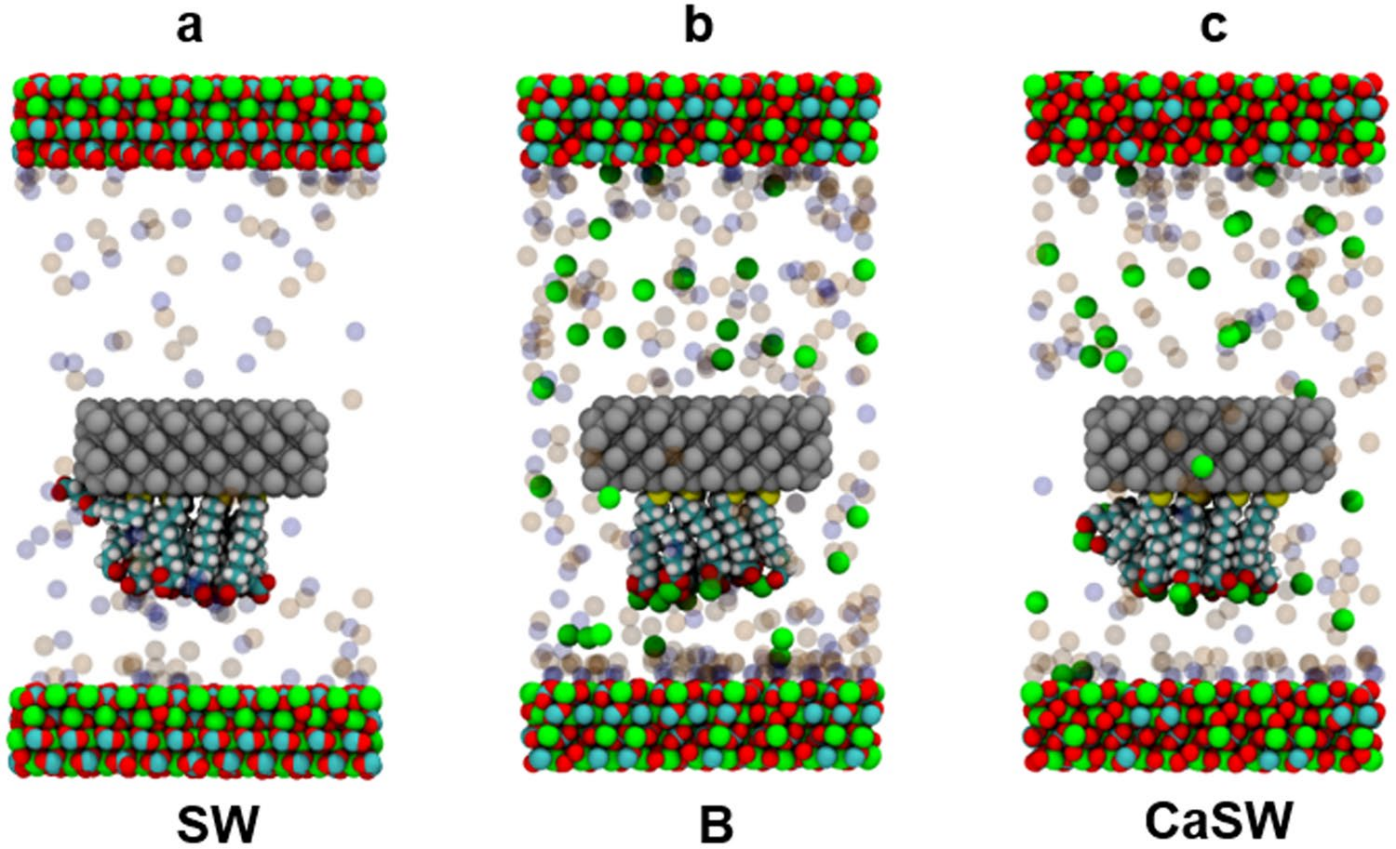
Snapshots of the simulation boxes after initial production runs with fixed silicon slabs in different reservoir fluids. In the fluids the ion colors are: Na^+^ = transparent blue; Cl^−^ = transparent orange; Ca^2+^ = green. Other atom colors are the same as in Fig. 1. Water molecules are removed for clarity. The alkanethiols in all three fluids start with similar order. The end carboxyl groups in seawater (SW) are surrounded by sodium ions, while those in brine (B) and calcium-doped seawater (CaSW) strongly chelate the calcium ions.

**Figure 4:**
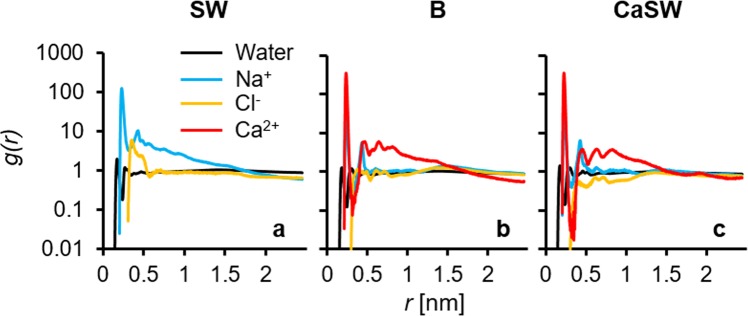
Radial distribution functions *g*(*r*) for the carboxylate groups with different ions or with water molecules in different reservoir fluids, confirming the complexation of the carboxylate groups by sodium ions in seawater (SW) and by calcium ions in brine (B) and calcium-doped seawater (CaSW).

First, the carboxylate and ion complexes were studied in the different reservoir fluids; seawater (SW), calcium-doped seawater (CaSW), and brine (B) as described in the methods section. Figure 3 shows the snapshots of the simulation boxes after initial 20 ns production runs with fixed silicon slabs. As can be clearly seen, when away from the calcite surface, the carboxylates strongly chelate the calcium ions in the B and CaSW fluids (Fig. 3b,c). Figure 4 shows the radial distribution functions *g*(*r*) for the carboxylate groups with different ions and with water molecules. In SW (Fig. 4a), the negatively charged carboxylate groups were mainly surrounded by the positively charged Na^+^ ions because of electrostatic interactions. However, in B and CaSW (Fig. 4b,c), the carboxylates bind the Ca^2+^ ions more strongly due to the chelating effect (c.f. Fig. 3b,c); the strong carboxylate-Ca^2+^ ion complexes have been well documented^44–46^.

**Figure 5:**
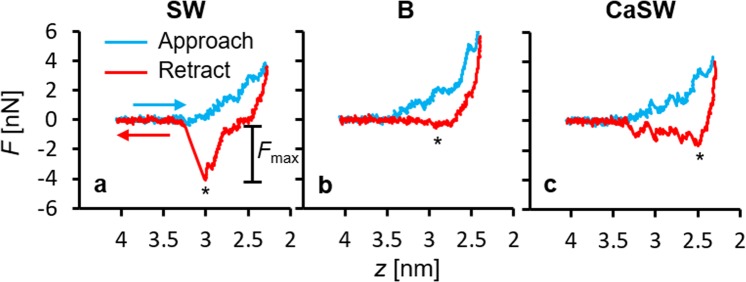
Representative force measurements from the steered molecular dynamics (SMD) simulations in different reservoir fluids. The blue curves are with the silicon slabs approaching to the calcite surfaces (decreasing *z*), and the red curves are with the silicon slabs retracting from the calcite surfaces (increasing *z*). The stars represent the maximum retracting forces, *F*_max_. The adhesion is much greater in seawater (SW) than in brine (B) or calcium-doped seawater (CaSW).

Next, steered molecular dynamics (SMD) simulations were used to model the AFM experiments where the adhesion force is measured as the minimum in the force curve as the simulated AFM tip is pulled away from the surface. Figure 5 shows the representative force measurements from the SMD simulations with the carboxylate-functionalized silicon slabs first approaching (blue curves; decreasing *z*) and then retracting from the calcite surfaces (red curves; increasing *z*). In SMD simulations the maximum force on the retraction curve, *F*_*max*_, represents the adhesion force measured experimentally by AFM, albeit not necessarily at the same order of magnitude. The behavior of the approach curves was similar in all fluids where the functionalized AFM tips experienced repulsion when the tethered alkanethiols are compressed against the calcite surfaces. The retraction curves, however, show variations with changing fluid chemistry where the functional groups in contact with the calcite surfaces experienced different adhesion forces in different fluids. As shown, the adhesion, marked by stars, was much stronger in SW (Fig. 5a) than in B and CaSW (Fig. 5b,c). This result matched nicely with prior experiments^40^.

**Figure 6:**
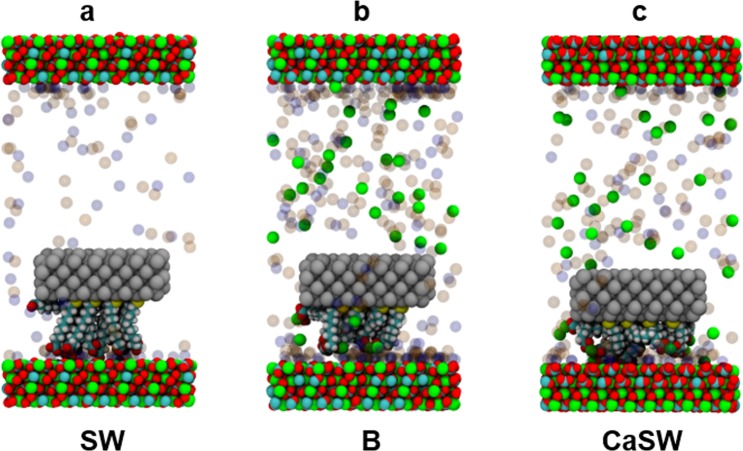
Snapshots of the simulation boxes at the maximum retracting forces (c.f. stars in Fig. 5) in different reservoir fluids. The atom and ion colors are the same as in Figs 1 and 3. Water molecules are removed for clarity. Note the difference in the order of the alkanethiols among the three fluids.

To explore this further, Fig. 6 shows the simulation snapshots when the functionalized AFM tips experienced the maximum retracting force (c.f. the stars in Fig. 5). As can be seen, in SW the carboxylates formed multiple direct contacts with the calcium ions on the calcite surfaces (Fig. 6a). In contrast, in B and CaSW, the strong complexes between the carboxylate groups and the Ca^2+^ ions in fluids prevented direct interaction between the carboxylate groups and their adhesion sites on the calcite surfaces (Fig. 6b,c). The force measurements were similar for the B and CaSW cases (Fig. 5b,c); nevertheless, there were microscopic differences. In the concentrated B fluids, a layer of NaCl can deposit on the calcite surfaces (Fig. 6b), which further screens the functionalized AFM tip interactions with the calcite surface interactions. This screening effect has previously been reported in Chen *et al*.^47^. On the other hand, in the less concentrated CaSW fluids, the functionalized AFM tips can still touch the calcite surfaces but the carboxylate groups were occupied by the calcium ions from solution and could not form strong adhesion to the calcite surfaces (Fig. 6c).

**Figure 7:**
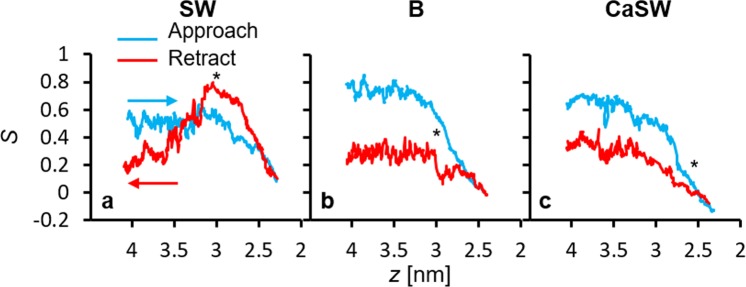
Representative traces of the order parameter, *S* = < (3 cos^2^*θ* − 1)/2>, where *θ* are the angles between the principal axes of the tethered alkanethiols and the pulling direction (*z* axis), from the SMD simulations in different reservoir fluids. Further examples are shown in the supplementary material. The blue and red curves represent the same sequences as in Fig. 5, and the stars represent the locations of the maximum retracting forces. While the change in order upon approach is similar for all three fluids, upon retraction in seawater (SW) the alkanethiols undergo the greatest variation in order.

In SMD simulations, we observed that the behavior of the tethered alkanethiols can be modified by specific ions. This phenomenon was captured by following the order parameter, *S* = < (3 cos^2^*θ* − 1)/2>, throughout the simulations. The order parameter is commonly used in studies of liquid crystals^48^, and *θ* are the angles between the principal axes of the tethered alkanethiols and the pulling directions (*z* axis) (Fig. 7). If S = 1, the alkanethiols will all be aligned with the *z* axis.

After initial equilibrium, prior to calcite-surface interactions, the alkanethiols were more aligned with the *z*-axis in B and CaSW (*S* ~ 0.7; Fig. 7b,c), while in SW the free-moving alkanethiols were less aligned (*S* ~ 0.5; Fig. 7a). The different behavior is because of the nature of the ions: in B and CaSW, a single calcium chelates with two carboxyl groups, restricting the motion of the alkanethiols, whereas in SW, sodium binds with only one carboxyl group. In SW, the alkanethiols are more mobile.

In the approach curves (blue curves in Fig. 7) for all fluids, *S* decreases with decreasing *z* due to alkane chain compression. For the retraction curves (red curves in Fig. 7, more examples shown in supplementary material), however, the *S* parameter behavior is distinctly different in SW compared to in B and CaSW. When retracting from calcite surfaces in SW, *S* first increased to ~0.7 at the maximum retracting force and then dropped to ~0.2 after the tip detached from the surface (Fig. 7a), whereas in B and CaSW, *S* changed much less dramatically (Fig. 7b,c).

**Figure 8:**
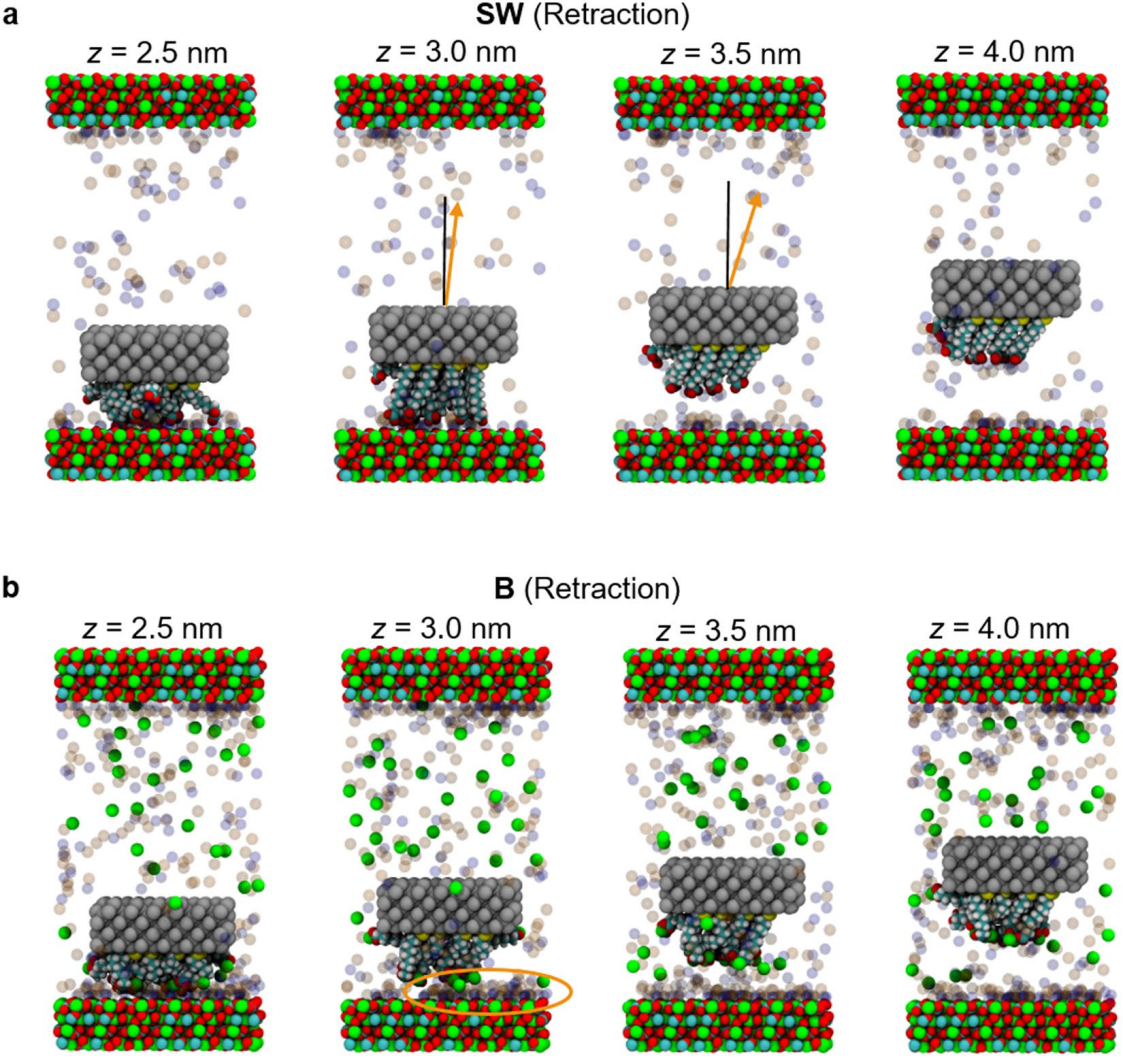
Simulation snapshots of the retraction sequences in SW and B. Note in SW the alkanethiols are nearly all aligned perpendicularly to the surface (S ~ 0.7) at *z* = 3.0 nm when stretched and show “spring back” morphologies at *z* = 3.5 and 4.0 nm. Similar behavior was not observed in the B and CaSW fluids. The orange arrows in the snapshots for SW (z = 3.0 and 3.5 nm) are the average orientations of the alkane chains before and after pull-off, and the orange oval in B (z = 3.0 nm) highlights the salt layer on the surface of the calcite.

Interestingly, the Ca^2+^ content affects the structure of the alkanethiols more on the retracting motion than on the approach. These results suggest that in SW the functional groups are bound tightly enough to the surface to promote rearrangement prior to detachment, which is not generally the case in the other two fluids. Figure 8 shows the retraction simulation snapshots in SW and B. As shown, the alkanethiols in SW have the highest alignment with stretched morphologies at the maximum retraction force (*z* = 3.0 nm), and then showed “spring back” morphologies after detachment from the calcite surfaces (*z* = 3.5 and 4.0 nm). This behavior was not observed in the other two fluids because the lower adhesion in these cases was not sufficient to stretch the alkanethiols prior to detachment, and therefore no subsequent “spring back” morphologies exist.

**Figure 9:**
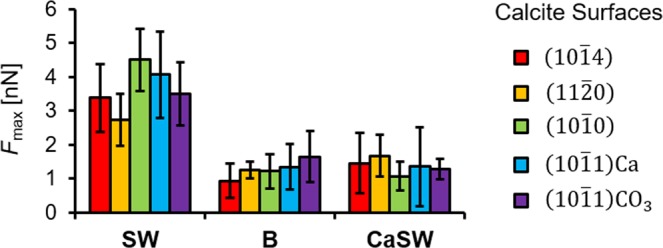
Summary of the maximum retracting forces, *F*_max_, in different reservoir fluids on different calcite crystallographic surfaces. The error bars are from five independent simulations in each case. Adhesion in seawater (SW) is consistently two or three times higher than in brine (B) and calcium-doped seawater (CaSW). There are small differences in adhesion among the five different crystallographic directions, with the (10 $\bar{1}$0) and (10 $\bar{1}$1)Ca surfaces showing higher adhesion than the other three.

The effects of calcite crystallographic structure on the maximum retracting force, *F*_max_, are shown in Fig. 9 for each fluid. The results showed that for all crystal structures the *F*_max_ values were about two to three fold higher in SW compared to in B and CaSW, which is quantitatively consistent with the previously reported experimental results^40^. It should be noted that the previous experimental studies and the current simulations are at room temperature and focused on the effects of calcium concentration on the measured interactions. Additional molecular dynamics studies were performed to demonstrate the effect of temperature and magnesium content separately (see Supplemental Information). These simulations show that the two fold increase is still observed at an elevated temperature of 100 °C (Fig. S2). In addition, simulation results in magnesium-doped seawater (MgSW) on selected calcite surfaces showed that, similar to the calcium ions, the magnesium ions can also mitigate adhesion but with lower efficiency (Fig. S3). These observations validate the predictability of the MD simulations, complemented the AFM experiments, and gave a more complete picture of this interesting calcium-mediated adhesion phenomenon in reservoir fluids.

**Figure 10:**
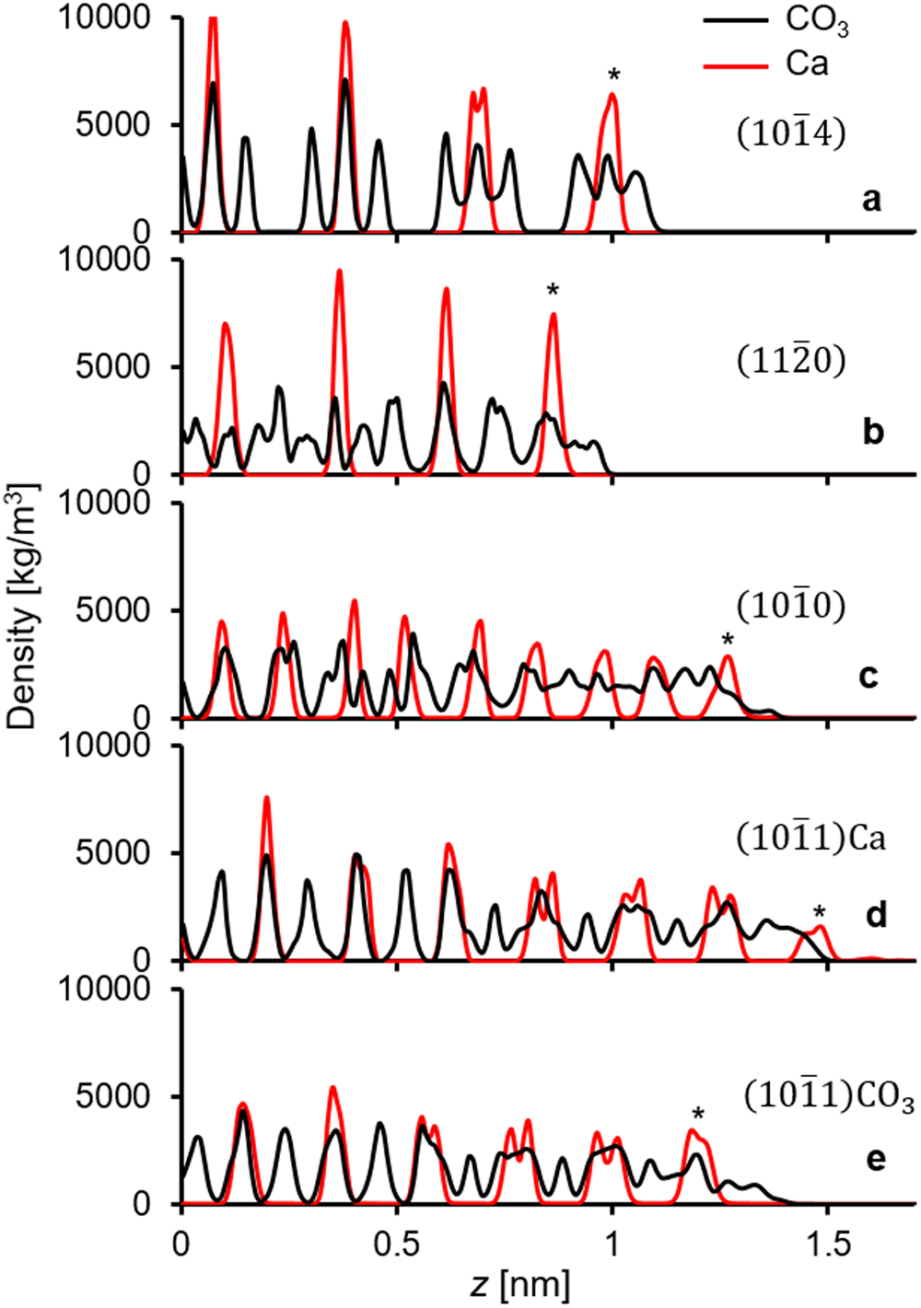
Density distributions of the calcite Ca (red) and CO_3_ (black) ions along the *z* axis. The stars represent the surface Ca ions that can interact with the carboxylate groups on the AFM tips. Consistent with the adhesion results for seawater in Fig. 8, calcium lies closer to the surface for the (10 $\bar{1}$0) and (10 $\bar{1}$1)Ca crystallographic faces.

Finally, the effects of calcium exposure for the various calcite crystallographic structures were examined. Figure 10 shows the ion distribution versus depth of the calcite surface for each crystallographic structure shown in Fig. 2. The exposed calcium ions on the calcite surface are marked with stars to indicate the ions available to interact with the carboxylate groups on AFM tips. In SW we observed that *F*_max_ was the highest on the (10$\bar{1}$0) surfaces and was the lowest on the (11 $\bar{2}$0) surfaces (Fig. 9). This is a result of the more accessible calcium ions on the (10 $\bar{1}$0) surfaces (c.f. Figs 2 and 10c) and the less exposed calcium ions on the (11 $\bar{2}$0) surfaces (Fig. 10b).

In summary, this work provides molecular-level details about the calcium-mediated adhesion of nanomaterials to calcite surfaces in reservoir fluids. Using atomistic molecular dynamics (MD) simulations, we first showed that the carboxylate groups on the AFM tips strongly chelate calcium ions in the B and CaSW fluids. We then performed non-equilibrium steered molecular dynamics (SMD) simulations to model an AFM experiment and measure the adhesion forces between the AFM tips and the calcite surfaces; the adhesion forces were two to three fold stronger in the Ca^2+^ deficient SW fluid compared to in the Ca^2+^ abundant B and CaSW fluids, which quantitatively matched prior experimental results^40^. Simulation snapshots directly show that the complexes between the carboxylate groups and the Ca^2+^ ions in solution prevented the direct carboxylate-calcite interactions, which resulted in decreased adhesion in the B and CaSW fluids. Inspecting the order parameter of the tethered alkanethiols on the AFM tips, we further discovered that the complexes between carboxylates and the Ca^2+^ ions reduce the alkanethiols’ initial mobility and that retracting the tip in SW aligns the alkanethiols prior to pull-off significantly more than in B or CaSW. SMD simulations on five different calcite surfaces showed that the adhesion forces were stronger in SW compared to in B and CaSW for all calcite crystallographic structures. Furthermore, in SW the calcite surfaces with more accessible calcium ions [e.g. (10 $\bar{1}$0) surfaces] had stronger adhesion compared to surfaces with less exposed calcium ions [e.g. (11 $\bar{2}$0) surfaces]. We believe the MD study put forward in this work not only captured the essential physics of the ion-specific nanomaterials adhesion in reservoir fluids, but also provided valuable molecular details that are experimentally inaccessible.

## Methods

The system studied consisted of a carboxylate-functionalized atomic force microscopy (AFM) tip analogue between extended calcite slabs (Fig. 1) in different reservoir fluids. The AFM tip analogue consisted of a square ~4 nm × ~4 nm silicon slab with 16 carboxylate-terminated alkanethiols (-S(CH_2_)_10_COO^−^) grafted with 0.54 nm grafting distances^49,50^. Five low surface energy calcite surfaces were considered (Fig. 2)^47,51–53^: (10 $\bar{1}$4), (11 $\bar{2}$0), (10 $\bar{1}$0), (10 $\bar{1}$1)Ca, and (10 $\bar{1}$1)CO_3_. Three reservoir fluids were considered in this study, of which the notations followed prior experiments^40^ but here the constituents were simplified for computational purposes: (1) seawater, SW: 0.7 M NaCl, (2) brine, B: 1.28 M NaCl + 0.34 M CaCl_2_, and (3) calcium-doped seawater, CaSW: 0.7 M NaCl + 0.34 M CaCl_2_. Notably, in this study we only focused on the NaCl and CaCl_2_ salts in order to pinpoint the calcium-mediated ion-specific behavior.

In the simulations, the calcite, calcite-water, and calcite-organic molecules interactions were described by the force field of Xiao *et al*.^54^, in which the organic molecules were described by the OPLS-AA force field^55^, and the water was treated with the TIP3P model^56^. The geometric combination rule was used to deduce all pairwise Lennard-Jones potentials from atom-wise CaCO_3_, OPLS-AA, and water model parameters. This force field has been previously used to study hydration layers as well as the carboxylate-calcite interactions with good compatibility^54,57^. The ions were modeled by the force field included in OPLS-AA parameter set originally developed by Åqvist and Chandrasekhar *et al*.^58,59^. Finally, the silicon slab was modeled by the GROMOS 53a6 force field^60^.

All molecular dynamics (MD) simulations were performed at temperature *T* = 300 K and *P* = 1 bar. Periodic boundary conditions were used in three dimensions, with an extended calcite slab perpendicular to the *z* axis. The lengths of the *x* and *y* box vectors were both ~5 nm, with the angle between *x* and *y* axis ranged from 90° to 113° depending on the terminated calcite crystallographic planes^47^. The space between the calcite slab and its periodic image were set to 7.5 nm, where the AFM tip was put in the middle (c.f. Fig. 1). In this work, MD simulations were carried out using GROMACS v2018.2^61^. Electrostatic interactions were calculated using the particle-mesh Ewald (PME) summation, with a real-space cutoff of 1 nm, a grid spacing of 0.16 nm, and fourth-order interpolation. The van der Waals and neighbor-list cutoffs were both set to 1 nm. We used velocity rescaling temperature coupling with a time constant of 0.5 ps and Berendsen semi-isotropic pressure coupling with a time constant of 5 ps. The simulation time step was set to 2 fs.

Before the production runs, the systems were pre-equilibrated with a steepest-descent energy minimization, a 0.1 ns *NVT*, and a 1 ns *NPT* simulations. The production runs consisted of three consecutive steps: (1) 20 ns equilibration run with restrained silicon slabs, (2) 40 ns non-equilibrium steered molecular dynamics^54,62–64^ (SMD) run with the silicon slabs approached to the calcite surfaces with constant velocity (0.05 nm/ns), and (3) 40 ns SMD run with the silicon slabs retracted from the calcite surfaces with constant velocity (0.05 nm/ns). The bottom layer of the calcite molecules was restrained in order to support the surface, while the top layer of the calcite molecules facing the AFM tip could freely move. All bonds with H-atoms were constrained using the LINCS algorithm^65^ with the exception of water molecules, which were constrained using the SETTLE algorithm^66^.

The order parameter was calculated as *S* = < (3 cos^2^*θ* − 1)/2>, where *θ* was the angle between the principal axes of the tethered alkanethiols and the pulling direction (*z* axis). The angle, *θ*, used in the order parameter was calculated during the SMD simulations by averaging the angles of the 16 tethered alkanethiols at every 100 ps interval.

## Supplementary information

Supplementary Material

## Data Availability

The data sets generated during and/or analyzed during the current study are not publicly available due to the corresponding author’s corporate affiliation but are available from the corresponding author on reasonable request.
