# Supplementary material for: Understanding Calcium-Mediated Adhesion of Nanomaterials in Reservoir Fluids by Insights from Molecular Dynamics Simulations
Source: Sci Rep. 2019 Jul 24;9:10763. doi: 10.1038/s41598-019-46999-8 (PMC6656760; doi:10.1038/s41598-019-46999-8)
Supplement: Supplementary file 1 — Supplementary Material [file 41598_2019_46999_MOESM1_ESM.pdf]

Supplementary Material for:

Understanding Calcium-Mediated Adhesion of Nanomaterials in Reservoir Fluids by Insights from Molecular Dynamics Simulations

Hsieh Chen[1,*], Shannon L. Eichmann[2], and Nancy A. Burnham[3]

[1]Aramco Services Company: Aramco Research Center-Boston, Cambridge, MA, 02139, USA

[2]Aramco Services Company: Aramco Research Center-Houston, Houston, TX, 77084, USA

[3]Physics and Biomedical Engineering Departments, Worcester Polytechnic Institute, Worcester, MA, 01609, USA

[*]hsieh.chen@aramcoservices.com

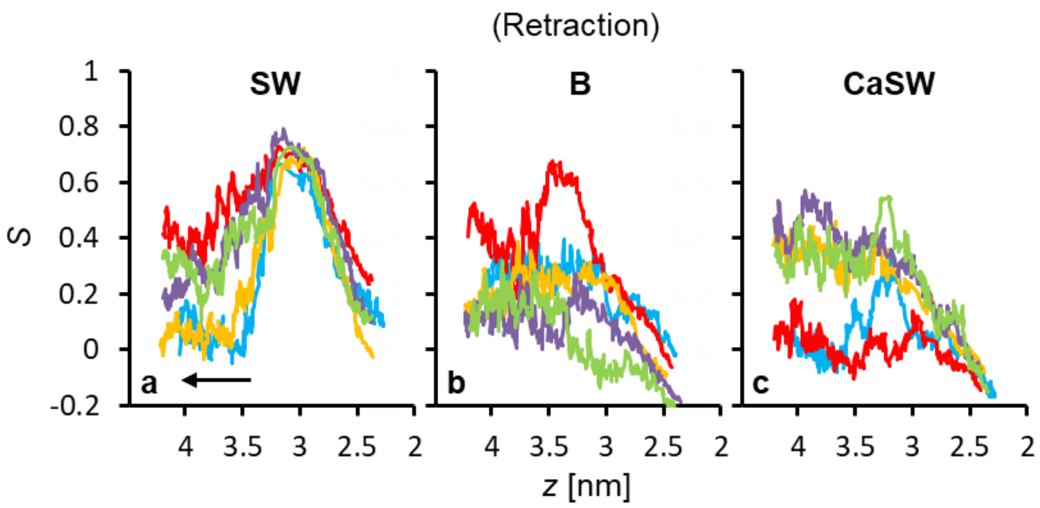

Figure S1. Further examples of the retraction curves of the order parameters, $S = \langle(3\cos^2\theta - 1)/2\rangle$, where θ are the angles between the principal axes of the tethered alkanethiols and the pulling direction (z axis), from the SMD simulations in different reservoir fluids.

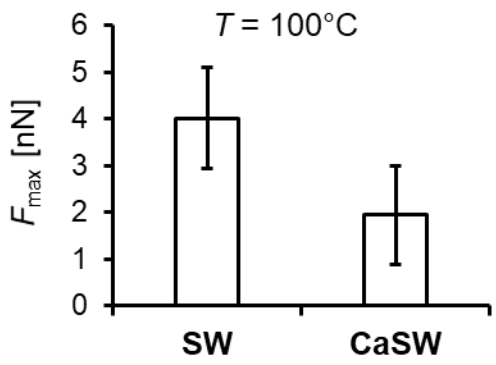

Figure S2. Maximum retracting forces, F_{max}, in seawater (SW) and calcium-doped seawater (CaSW) on calcite (10$\bar{1}$4) surfaces at elevated temperature T = 100 °C. The error bars are from five independent simulations. Adhesion in SW is about two times higher than in CaSW at this temperature.

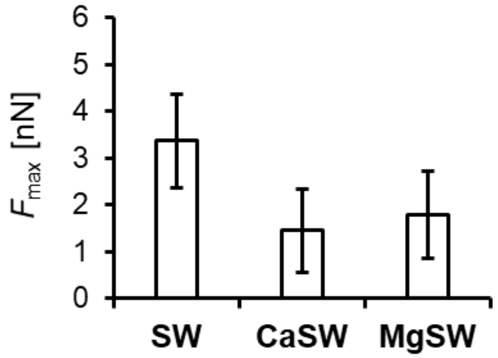

Figure S3. Maximum retracting forces, F_{max}, in seawater (SW), calcium-doped seawater (CaSW), and magnesium-doped seawater (MgSW) on calcite $(10\bar{1}4)$ surfaces. The error bars are from five independent simulations in each cases. The data suggested that the magnesium ions can also mitigate adhesion but with lower efficiency compared to the calcium ions.
